# Psychiatric issues in renal failure and dialysis

**DOI:** 10.4103/0971-4065.42337

**Published:** 2008-04

**Authors:** A. De Sousa

**Affiliations:** Consultant Psychiatrist, Get Well Clinic, Mumbai, Maharashtra, India

**Keywords:** Dialysis, psychiatric aspects, renal failure

## Abstract

This article aims to bring to the fore, issues regarding the interface of psychiatry and renal failure. Depression, anxiety, suicide and delirium are common complications observed in patients with renal failure. Pharmacological management of these problems need stringent monitoring on part of the psychiatrist. This article examines the various complications that may be observed in patients with renal failure while discussing treatment approaches and also emphasizing the need for interdisciplinary team work in improving the quality of life of patients with renal failure and those on dialysis.

## Introduction

Chronic kidney disease is a multifaceted problem having both physical and psychological connotations for the patient. A multidisciplinary team effort is often needed in the management of such patients. Mental health professionals may need to collaborate with nephrologists for a holistic management of such patients. The patients suffering from renal failure often present with unusual psychological problems where treatment methods vary on an individualized basis and drug therapy is often needed in the management of such problems.

Patients on dialysis are in a situation of abject dependence on a machine, a procedure and a group of qualified medical professionals for the rest of his/her life.^1^ No other medical condition has such a degree of dependence for the maintenance treatment of a chronic illness.^2^ Dialysis as a procedure is stressful for the patient in the event of inadequate education and preparation with regard to pre-end-stage renal disease (ESRD). There is also a considerable restraint on the selection of foods and fluids. Patients on peritoneal dialysis have some latitude regarding this compared to patients on hemodialysis.

Patients with renal failure often suffer from many other medical conditions and are on many different medications. Many of these medications may at times cause psychiatric symptoms and it is worth noting the same to avoid confusion [Table 1]. Sometimes agitation and confusion may be noted as a result of nonpsychiatric medication. These are very perplexing symptoms since the same may be observed in medical conditions such as electrolyte disturbances, hypertension, hypoglycemia, aluminum toxicity, dialysis dementia and may also be a part of depression and anxiety.^3^

**Table 1:** Selected drugs associated with neuropsychiatric morbidity and their implications on patients with renal failure

Drug	Symptoms	Accumulation in ESRD
Amantadine^13^	Agitation, anxiety, confusion, depression, hallucinations and insomnia	Yes
Aspirin^14^	Anxiety, insomnia, confusion, psychosis	No
Ciprofloxacin^15^	Agitation, psychosis	Yes
Diphenhydramine^16^	Confusion, anxiety, sedation	Probable
Interferon^17^	Confusion, psychosis depression, fatigue, suicidal tendencies, irritability	Not studied
Pentazocine^18^	Confusion, hallucinations	Yes
Phenytoin^19^	Confusion, dizziness, drowsiness, insomnia	Yes
Prednisolone^20^	Anxiety, insomnia, psychosis	No

## Depression

The most common psychiatric complication occurring as a result of renal failure is depression in the patient and anxiety in the associated partner.^3^,^5^ Most dialysis patients who are employed may seldom return to full time work activity. Work in addition to a source of income is often associated with a sense of accomplishment, self-esteem and identity in most patients. The current accepted psychiatric treatment for depression would include an antidepressant therapy combined with psychotherapy. Special considerations are needed while putting ESRD patients on antidepressant therapy. Presently, a wide variety of antidepressant drugs are available for the management of depression. Each of these may have varied effects on renal function, although most are safe in a large number of cases. Comparative doses of these drugs in normal adults and in those with renal failure, including their pharmacological class and side effects, have been reported [Table 2]. No studies so far have compared depression in patients with hemodialysis and continuous ambulatory peritoneal dialysis (CAPD); however, reviews with regard to the occurrence of depression in renal failure patients clearly mention that patients on CAPD experience milder symptoms.^3^

**Table 2:** Antidepressant drugs and renal function

Drug	Normal adult dose	Dose in renal failure	Comment
Selective serotonin reuptake inhibitors (SSRIs)
Citalopram^21^	20-60 mg	10-60 mg	Very safe. Fluoxetine may cause loss of appetite at times and insomnia as a side effect
Escitalopram^22^	10-20 mg	5-20 mg
Fluoxetine^23^	20-60 mg	20-40 mg
Fluvoxamine^24^	50-300 mg	50-300 mg
Sertraline^25^	50-150 mg	50-150 mg
Paroxetine^26^	20-60 mg	10-30 mg	Dose should be reduced in renal failure May cause seizures at high doses
Tricyclic antidepressants
Amitryptiline^27^	25-75 mg	25-75 mg	Safe but side effects like constipation, dry mouth, blurred vision. Trazadone may cause priapism at high doses
Imipramine^27^	25-75 mg	25-75 mg
Doxepin^28^	25-75 mg	25-75 mg
Amoxapine^27^	75-200 mg	75-200 mg
Nortryptiline^27^	25-75 mg	25-75 mg
Trazadone^29^	150-400 mg	150-300 mg
Newer antidepressants
Venlafaxine^30^	37.5-225 mg	37.5-225 mg	May increase BP at high doses
Mirtazapine^31^	15-45 mg	7.5-30 mg	Sedation common
Duloxetine^32^	10-80 mg	10-80 mg	Safe

## Suicidal Behavior

Discussing depression further brings up the subject of suicidal behavior in dialysis and renal failure patients. Repeated observational studies have demonstrated that dialysis patients have higher suicide rates than the normal healthy population.^6^ It is noteworthy that when depressed, the dialysis patient has at his disposal a very effective method of escape i.e. suicide. Simply missing dialysis for some sessions or going on a potassium food binge can produce death. Moreover, under consideration in case of suicide would be the voluntary withdrawal from dialysis and ethical issues involved in it, which are beyond the scope of this article.

## Delirium

Delirium is a common phenomenon observed in dialysis patients due to electrolyte imbalances that may occur after a dialysis run termed as the dialysis disequilibrium syndrome or as a consequence of medical or surgical complications.^7^ The causes may include uremia, anemia and hyperparathyroidism. In any aging population having diabetes and receiving dialysis, dementia may occur due to Alzheimer's disease, vascular causes and dialysis dementia syndrome. The latter is a progressive disorder and is often fatal. In all cases, the management would be on a case-by-case basis and early diagnosis and detection is a must. Ameliorative medications such as antipsychotics, lorazepam and neurotropics may be useful in these conditions. Information regarding their use in renal failure patients is sparse, and the drug of choice is often the result of past successful experiences. A list of these drugs and their normal doses along with dose restrictions in patients with renal failure has been provided [Tables 3 and 4].

**Table 3:** Drugs used in delirium in renal failure

Drug	Dose in normal adults	Dose in renal failure	Comments
Haloperidol^34^	5-15 mg	5-15 mg	May increase QT interval
Clozapine^35^	25-400 mg	Titrate dose as needed	May cause seizures above at 400 mg Agranulocytosis is common
Olanzapine^36^	5-20 mg	5-20 mg	Safe
Quetiapine^37^	150-600 mg	150-600 mg	-
Risperidone^38^	1-4 mg	0.5-2 mg	Sedation
Ziprasidone^37^	20-80 mg	20-80 mg	May increase QT interval
Piracetam	800-4800 mg	800-4800 mg	Safe

**Table 4:** Anti-anxiety drugs and renal failure

Drug	Dose in normal adults (mg/day)	Dose in renal failure (mg/day)	Comment
Alprazolam^38^	0.25-4 mg	0.25-2 mg	Effective in the management of anxiety and insomnia. May cause drowsiness at high doses that may be confused with delirium
Clonazepam^39^	0.5-1.5 mg	0.5-1.5 mg
Lorazepam^39^	1-4 mg	1-4 mg
Diazepam^40^	5-40 mg	5-25 mg
Buspirone^41^	5-20 mg	5-20 mg	Safe anti-anxiety drug. No sedation
Zolpidem^42^	5-20 mg HS	5-20 mg HS	Short-acting drugs and no residual drowsiness
Zaleplon^42^	5-10 mg HS	5-10 mg HS

## Anxiety and Panic Symptoms

Extreme anxiety and anxiety somatic symptoms such as breathlessness, palpitations, chest pain, sweating and fear of dying may occur in renal failure cases. Many a times, these symptoms are not associated with any triggers and may occur unexpectedly. There are, on the other hand, many reasons regarding the occurrence of anxiety. The process of dialysis and a multitude of potential medical complications give the patient a lot to worry and anticipate about. Pharmacological management is paramount in the management of anxiety and panic. Benzodiazepines such as clonazepam and alprazolam may be used to reduce anxiety in such patients. Many patients with anxiety also tend to experience insomnia. Drugs such as zolpidem and zaleplon are useful in the management of such insomnias with no residual drowsiness and minimal side effects. Doses of some of these drugs may have to be adjusted to suit the needs of the nephrologically compromised patient [Table 4]. One must be careful with benzodiazepines as a group as they tend to cause sedation at higher doses which may be mistaken for other delirious conditions.

## Problems in Receiving Psychiatric Help

Renal failure patients have been noted as the biggest deniers of psychiatric illness.^1^ They often feel that they are overdoctored and even motivational psychotherapy is best administered in the dialysis unit itself. Many patients on dialysis do well if individual psychotherapy is administered during the dialysis sessions itself. Another complication is the nonadherence to the treatment and medical regimens. Such patients take appointments, but do not visit the doctor and may also get angry on the staff of the dialysis unit. The dialysis population is not just a cross section of the general population. This group is skewed in the direction of the noncompliant diabetic, noncompliant hypertensive and also the alcoholic. These patients often express their anger as they feel that many others lead a normal life, while they have to suffer and undergo repeated medical procedures.

Professional staff members handling dialysis patients are high achievers in life educationally and professionally. They often have the tendency to identify with patients and project their values regarding dialysis on the patients regarding what they would do if they had renal failure and had to undergo dialysis. This is a common fallacy and needs to be kept in mind while setting realistic goals for these people. The staff members must be strict while handling patients, although gentle when needed.

The average age of the ESRD patient is 65 years. They are not only older but more infirm.^8^–^9^ Many of them have comorbid diabetes, hypertension, peripheral arterial disease, cardiomyopathies and arthropathies, which contribute to patient symptoms and reduction in the quality of life (QOL).^10^ȓ^11^ Denial of death is also a common problem in such patients. Mental health professionals may often be needed in such cases to provide end-stage counseling and psychotherapy.^12^

An increase has been projected in ESRD patients worldwide with decrease in the number of nephrologists, specialized staff and professionals trained to help them. An effective team work often, interdisciplinary, is a must in the effective management of interconnected problems; it is only the collaborative effort that leads to better outcomes and improved quality of life.^41^
